# Structural and Functional Analyses of Cone Snail Toxins

**DOI:** 10.3390/md17060370

**Published:** 2019-06-21

**Authors:** Harry Morales Duque, Simoni Campos Dias, Octávio Luiz Franco

**Affiliations:** 1 Centro de Análises Proteômicas e Bioquímicas, Programa de Pós-Graduação em Ciências Genômicas e Biotecnologia, Universidade Católica de Brasília, Brasília-DF 70.790–160, Brazil; hamorales30042033@gmail.com (H.M.D.); si.camposdias@gmail.com (S.C.D.); 2 S-inova Biotech, Programa de Pós-Graduação em Biotecnologia, Universidade Católica Dom Bosco, Campo Grande-MS 79.117–900, Brazil

**Keywords:** cone snails, conotoxins, ion channels, function, structure

## Abstract

Cone snails are marine gastropod mollusks with one of the most powerful venoms in nature. The toxins, named conotoxins, must act quickly on the cone snails´ prey due to the fact that snails are extremely slow, reducing their hunting capability. Therefore, the characteristics of conotoxins have become the object of investigation, and as a result medicines have been developed or are in the trialing process. Conotoxins interact with transmembrane proteins, showing specificity and potency. They target ion channels and ionotropic receptors with greater regularity, and when interaction occurs, there is immediate physiological decompensation. In this review we aimed to evaluate the structural features of conotoxins and the relationship with their target types.

## 1. Introduction

Cone snails are marine mollusks from the Conidae family (Fleming, 1822 sensu lato), divided among 152 genera and involving 918 species described until now [1]. They are predatory carnivores that compensate for their slow movement by using hunting strategies with an arsenal of toxic peptides [2]. These molecules are known as conotoxins or conopeptides, with a wide variety of molecular masses ranging from conopressin-S with nine [3], to conkunitzin-S1 with 60, amino acid residues in length [4]. Due to their targets (i.e., Na^+^, K^+^, and Ca^++^ channels; ligand-gated ion channels; G-coupled proteins; and neurotransmitter transporters), conotoxins produce diverse physiological alterations, principally in excitable tissues [5]. These conotoxins are employed by cone snails to target their prey such as marine worms, snails and fish [6]. Because of the diverse species groups targeted by cone snails, their conotoxins need to act on specific targets of each species (e.g., subtype of ion channels) [7]. As previously studied, cone snails developed their toxins for hunting, but humans are not natural prey for them. However, accidents caused by cone snails’ sting have resulted in human injuries, which have been lethal in some cases [8]. Thus it has been demonstrated that *Conus* spp. venom has toxic compounds that also act on specific mammal transmembrane proteins [5]. Transmembrane proteins, such as ion channels or ionotropic receptors, are responsible for basic neurotransmission or signal transduction, which triggers other physiologic functions [9,10]. When these transmembrane proteins are affected, multiple human diseases arise [11,12,13]. These ion channel disorders are sometimes called channelopathies [14]. The natural capability of conotoxins to target these objectives could be used for disease treatment [15]. Therefore, the pharmacological properties of conotoxins have become a valuable biotechnological tool for potential drug development [16,17]. 

Conotoxins are structurally variable in reference to their function [18]. Recently, conotoxin classification was addressed by categories (i.e., by gene superfamily or pharmacological family, by cysteine (Cys) framework and connectivity, by loop class, by fold and subfold classes) [19]. The superfamily group was classified based on the nucleic acid sequence from the toxin’s signal peptides’ identity [20]. Conotoxin cDNAs have been grouped into 41 different superfamilies (Table S1) [21]. The family classification is based on the target type and action mode of conotoxins, independently of their structural features [5]. The present review uses this categorization (Table 1).

Structurally, conotoxins are diverse and categorized by their mature peptide [20,22]. They can be linear peptides without a disulfide bond, like the conantokins [23], or may possess between one and five disulfide bonds [5]. Those with multiple disulfide bonds adopt special three-dimensional conformation due to a different Cys distribution pattern (framework) in the toxin sequence (Table S2) and Cys pair connectivity type [18]. Loop class is a category used to divide the α-conotoxin family into subclasses [24]. This subclassification will be further explained (see α-conotoxin section below) (Table 2). For conotoxins with disulfide bonds, characteristics such as the cysteine framework, their pair connectivity, and number of amino acids provide them with a fold structure that favors their activity [18,19,25]. Conotoxins’ structural properties seem to be important for target interaction [25]. Importantly, other features are present in natural conotoxins such as the location of key amino acids on their primary sequence [5] and post-translational modifications, including amidation, sulfation, pyroglutamylation, γ-carboxylation, hydroxylation, O-glycosylation, and bromination [26]. On the whole, these features must be considered when analyzing conotoxin/target interaction. 

The structure/function diversity in conotoxins makes research a challenge [27]. Computational analysis has been used improve the cost/benefit ratio in conotoxin studies [28], trying to solve this problem. Due to the variable nature of conotoxins, there is no consensus that allows the mentioned categories to be linked with the family classification (see Table S3) [29]. The distance between the activity and structural variability of conotoxins makes investigation complex [21]. However, it is deduced that for each target there is one conotoxin that has greater potency and affinity than others of its type. In the following section, conotoxin families will be briefly mentioned, emphasizing that these toxins are completely characterized by interaction with their target. 

## 2. Conotoxin Families

With some exceptions, conotoxins are commonly named following a convention [30]. First, a Greek letter makes reference to a family in pharmacology (e.g., α, µ, κ, ω, etc.); the next two letters indicate the initials of the Conus species (e.g., Cg = *Conus geographus*), followed by one Roman number referring to the Cys framework (e.g., I, II, III, IV, etc., as shown in Table S2) and, finally, one uppercase letter indicates the discovery order (e.g., A, B, C, etc.) [18]:

αCgIA

These polypeptides have been divided into families by their pharmacological function [31]. Table 1 shows different family groups indicated by one Greek letter. Among them, the α-conotoxin family is distributed among different Conus species [32]. This toxin group is the most studied [23]. Other toxin groups—the µ-, ω-, and κ-conotoxins—have been the most characterized [33]. In general, some toxins from these family groups show special characteristics than allow specific interaction with their respective target group [5]. Thus, the following sections will describe conotoxins’ activity in different ion channels, with special attention to those conotoxins best characterized.

## 3. Conotoxins Interacting on Nicotinic Acetylcholine Receptors (nAChRs) 

**Figure 1:**
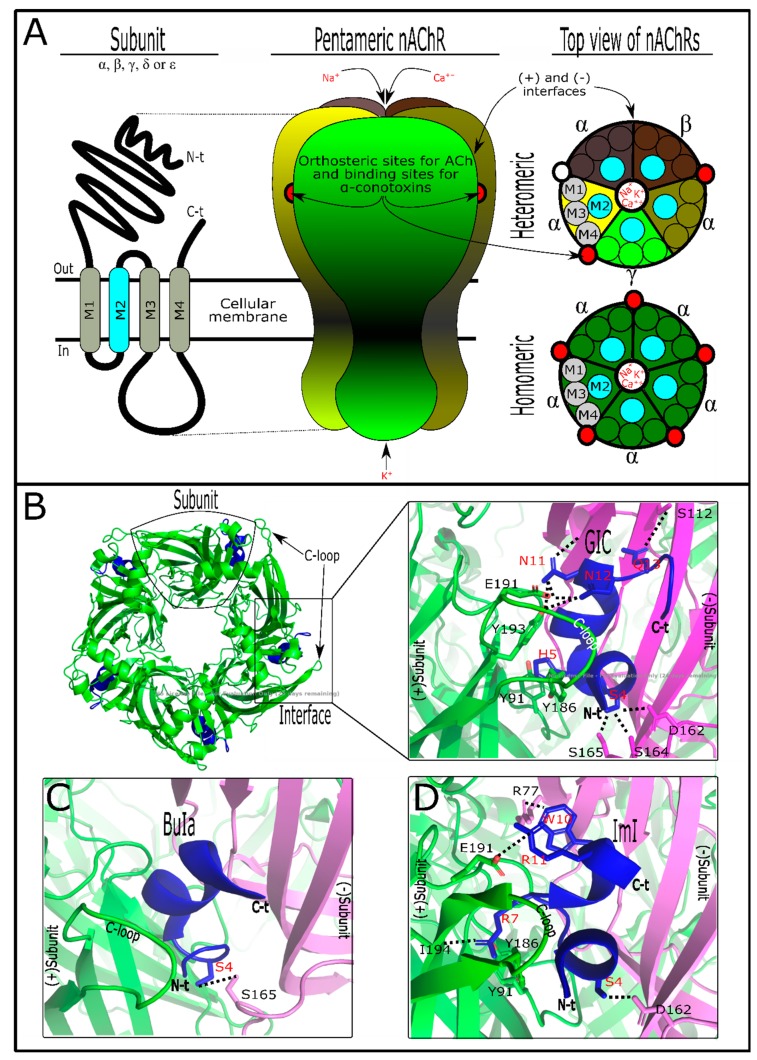
nAChRs and α-conotoxin interactions. (**A**) Basic subunits and pentameric structures of nAChRs. In the “top view” the segments’ organization is shown, with segment 2 forming the ion pore. nAChRs could be composed of different subunit types (heteromeric) or identical α-subunits (homomeric). The subunit junctions are the interfaces. (**B**) GIC and Ac-AChBP interaction (PDB: 5CO5) showing top view of this complex. On the left, α-conotoxin GIC (blue) is fitted into the Ac-AChBP interfaces. On the right, specific interaction points between GIC and Ac-AChBP are showed. C-loop from (+)subunit is highlighted. (**C**) Specific interaction points between BuIa and Ac-AChBP are showed (PDB: 4EZ1). (**D**) Specific interaction points between ImI and Ac-AChBP are showed (PDB: 2C9T). Amino acid residues in α-conotoxins are showed in red. Note that α-conotoxins are similarly oriented when they interact with their targets. nAChR structures were designed as described by the authors in the text.

Nicotinic acetylcholine receptors (nAChRs) (Figure 1A) are pentameric structures (five subunits surrounding one central filter that allows the flow of Na^+^, K^+^, and Ca^++^ ions) in which each subunit is composed of four transmembranal segments [45]. There are different subunit types such as α, β, γ, δ, and ε, which can form homomeric (identical subunits) or heteromeric (combination of subunits) nAChRs [46]. These ligand-gated ion channels, expressed in both the nervous system and non-neuronal cells, have a varied number of ligand sites for acetylcholine (ACh) depending on the nAChR subtype [47]. In the nervous system, nAChRs are involved in physiological functions such as analgesia, learning, memory, arousal, and motor control [45], while in the non-neuronal cells, nAChRs promote cell proliferation, secretion, migration, survival, and apoptosis functions [48]. When nAChRs from the nervous system are affected, they generate neuronal disorders such as cognitive disorder, depression, anxiety [11], epilepsy, pain, and diseases, including Parkinson’s and Alzheimer’s [47]. As seen, nAChRs have been demonstrated to be involved in multiple physiological processes, depending on the nAChR-specific subtype responsible for each activity [49]. Curiously, α-conotoxins have shown target nAChR subtypes [30,34]. They are antagonist competitors from acetylcholine binding sites [50]. As a result, α-conotoxins have become an important research tool to analyze interaction with nAChRs [51]. 

α-Conotoxins are diverse in structure and have been subclassified by loop class [24]. In this respect, the classification may be based on amino acid number distribution among Cys (C_1_C_2_*m*C_3_*n*C_4_), in which *m* and *n* are loops, where *n* is a defined number (3/m, 4/m, and 5/m) and *m* is a variable number of amino acids from each α-conotoxin type [18]. However, this classification could be applied for those α-conotoxins from the A superfamily with type I framework (Table 2 and Table S2), which possess Cys (C_1_–C_3_ and C_2_–C_4_) globular connectivity [51]. Alternatives for folding (C_1_–C_4_ and C_2_–C_3_ and C_1_–C_2_ and C_3_–C_4_) of these synthetic toxins are named ribbon and beads, respectively (Figure S1A) [52]. α-conotoxins from other superfamily groups with different frameworks are not included on this list [30]. Below, the structural diversity of α-conotoxins is shown for clarification. These structures allow them to be specific for their target group (Table 2 and Table 3). Some α-conotoxins (3/5) are specific for muscle nAChR subtypes, while other (4/3, 4/4, and 4/7) groups are selective for neuronal nAChR subtypes [53]. However, these toxins have shown promiscuous activity in different neuronal nAChR subtypes (Table 3) [51]. The promiscuity of α-conotoxins could be beneficial for these mollusks as a biological function [32], but it is disadvantageous for pharmacological purposes. Thus they need to be re-engineered for development as target-specific tools [54].

The capability of α-conotoxins to differentiate between neuronal and muscle nAChRs is due to the subunits’ composition in these receptors. Neuronal nAChRs can be homomeric when structured by α-subunits of the same type (α7, -8, or -9) or heteromeric when the α-subunits (α2-10) are combined together or with β-subunits (β2 and β4) [49]. In contrast, muscle nAChRs combine with α1, β1, γ, δ, and ε subunits [46]. They are not shared between tissues, and each subunit possesses its particular feature. So, structurally, α-conotoxins can discriminate among them [63]. Acetylcholine has orthosteric binding to the interface between nAChR subunits [45]. The acetylcholine affinity for these binding sites is due to hydrophilic features from each subunit’s nAChR composition [46]. In this way, homomeric subunits (composed of five identical α-subunits) have five binding sites, while heteromeric nAChRs commonly have two binding sites (Figure 1A), in some cases with an accessory binding site for acetylcholine [45,49]. 

These events have shown differences in activity responses between target nAChR subtypes. It was demonstrated that the acetylcholine concentration in two nAChR isomers, one with double orthosteric interaction points and the other with an additional binding site, stimulated a second phase of macroscopic currents in that nAChR with the third interaction point [64]. Similarly, α-conotoxins interact in nAChRs as antagonist competitors [50]. They bind with diverse affinities on the subunit interfaces, which can vary depending on subunit composition [53]. The variable combination of subunits in nAChRs could explain the promiscuous nature of α-conotoxins (Figure 1A, Table 3). Nevertheless, a certain selectivity of some mutated α-conotoxins in favor of nAChRs containing α3-, α6-, α7-, and α9/α10 interfaces, but not for α2- and α4-interfaces, has been observed [30]. As with acetylcholine, α-conotoxins can interact on various ACh binding sites (i.e., 2, 3, or 6) simultaneously with the same nAChR or homologous AChBP [50,65]. However, it was suggested that only one molecule is enough to inactivate nAChRs [66,67]. Thus, the α-conotoxin/nAChR isoform’s stoichiometric interaction could interfere in the real potency of toxin metrics.

Natural α-conotoxins have post-translational modifications (Figure S1B), with C-terminal amidation being the most frequent [63]. Post-translational modifications such as this allow biological activity in these molecules [68]. However, it is not a general rule. It was shown that the presence/absence of sulfated tyrosine in α-AnIB and EpI toxins has no significant biological difference [50,62]. More interestingly, this post-translational modification and the C-terminal amidation together could favor the affinity of α-AnIB for α7 nAChR [62]. These facts show that post-translational modifications in α-conotoxins can interfere in conotoxin/nAChR interactions. Indeed, it was suggested that amidation promotes native folding in this toxin group, leading to their selectivity [26]. Previous experiments showed that the proline from the *m*-loop and C-terminal amidation in α- and χ-conotoxins act as conformational switches [69]. With some exceptions (Figure S1B), the *m*-loop from α-conotoxins possesses one serine followed by one proline [63]. Proline facilitates α-helix formation, while serine provides a hydrophobic patch in these conotoxins’ loop [54]. This hydrophobic patch from α-conotoxins could interact with any subunit (-) interface pocket because the nAChR subunits in this site are not very hydrophilic [46]. The (-) here is specified as the subunit receptor interface of anything other than an nAChR α-subunit (Figure 1). In fact, it was suggested that when α-conotoxins interact with their target, they are directionally positioned in a similar manner independently of their amino acid sequence [30]. Similarly, the second loop, the *n*-loop, appears to be involved with the (-) subunit interface due to the presence of key amino acids in α-conotoxins responsible for the interaction [70]. However, the key amino acid in the fifth position of α-conotoxins is important for the (+)α-subunit interface interaction [70]. The (+)α-subunit interface pocket site is more hydrophilic [46] and possesses the C-loop, which plays a significant role in α-conotoxins’ interaction [54]. 

In some cases, the tertiary structure has been shown to be relevant in α-conotoxins’ activity, e.g., the ribbon isomer from native AuIB was seen to be 10-fold more potent in α3β4* nAChRs [50]. Pu1.2, Pn1.2, and Vc1.1 isomers, separately, demonstrated similar activity regarding their targets [52]. The tertiary structure is very important in the function of α-conotoxins because it leads to the spatial amino acid arrangement [18]. Additionally, for an electrostatic surface [18], this offers a special toxin three-dimensional shape that allows it to fit into the nAChR pocket binding site (Figure 1B). C-loops, the local binding site in nAChRs, are considered to be flexible, acting as a hinge that allows the toxin to fit into pocket interface subunits [54,81]. C-loop flexibility is conditioned by amino acid composition, which varies between nAChR subunits [54]. So, α-conotoxin size and shape are important for their interaction. However, α-conotoxins have been shown to be unselective [54]. This probably occurs because the electrostatic surface is able to interact with the nAChR pocket subunits’ interface [18]. A basic explanation of this specific phenomenon is that it could occur due to the similarities between the allosteric sites of different receptor subtypes [82]. Interestingly, it was suggested that nAChR orthosteric sites (acetylcholine) are notably conserved among organisms from different taxonomic groups [83].

α-Conotoxins have key residues that recognize their targets’ counterparts [54]. These amino acids were detected by scanning mutation [70]. Mutations were developed to enhance the toxin’s activity or selectivity [54]. However, this review will only mention the interactions of natural toxins and their targets. As previously mentioned, α-conotoxins show the same spatial position pattern when acting on targets [30]. This is due to the similar point-by-point connection between α-conotoxins and targets [70]. Natural α-conotoxins GIC, BuIA, and Iml, for example, show the characteristics of these interactions (Figure 1B–D). As observed, α-conotoxins recognize the key interaction point of acetylcholine receptor subtypes which are localized in (-) and (+)subunits’ interfaces [70]. GIC has the Ser4 common to α-conotoxins [72]. Ser4 and Gln13 interact by hydrogen bond with (Asp162, Ser164, or Ser165) and Ser112 from Ac-AChBP (-)subunits’ interface, respectively [84], while residues such as His5 and (Asn11, Asn12) interact with (Tyr91, Tyr186) and (Glu191, Tyr193) from Ac-AChBP (+)subunits’ interface, respectively [84]. As happens with Ser4 in α-conotoxins, Asn11 and Asn12 are shared between some of these toxin groups [85] and shown to be significant for toxin interaction on loop C, localized at Ac-AChBPs (+)subunits’ interface [70]. By alanine scanning mutation, it was observed that GIC has important residues for interactions with Ac-AChBPs or hα3β2, but not for interactions with hα3β4 subtypes [84,86]. GIC (Gln13Ala) did not appear to be relevant in the interaction with Ac-AChBPs or hα3β2 subtypes [84]. However, Gln13 produced a steric clash on Arg108 from (-)subunits hβ4, preventing affinity with hα3β4 subtypes [84]. These differences are shown in the comparison of acetylcholine receptor subtypes’ alignment (Figure S1C)

Similarly, BuIa shares Ser4, which is typical of α-conotoxins [57]. This serine makes a hydrogen bond with Ser165 from Ac-AChBPs (-) subunits’ interface, while other residues do not seem to be significant for Ac-AChBPs interaction [70]. However, BuIa shows affinity for neuronal nAChRs containing rα6/α3 and β2 subunits, more than for rα4 or β4 subunit interfaces [57]. Lys185, Thr187, Ile188, Thr198, and Tyr205 from rα6 (+)subunits’ interface were seen to be responsible for BuIa interaction [87]. Another α-conotoxin, ImI, was active in homomeric α7 and α9 nAChRs [74,88]. Ser4 from ImI, like GIC and BuIa, is localized spatially, allowing it to interact with Asp162 from the Ac-AChBPs (-)subunit interface [70]. Trp10 of ImI makes contact, by a hydrogen bond, with Arg77 from the same AChBP interface [89]. Arg7 and Arg11 contact (Tyr91, Tyr186, and Ile194) and Glu191 from the Ac-AChBP (+)subunit interface, respectively [70,89].

## 4. Conotoxins Interacting in Potassium Channels

**Figure 2:**
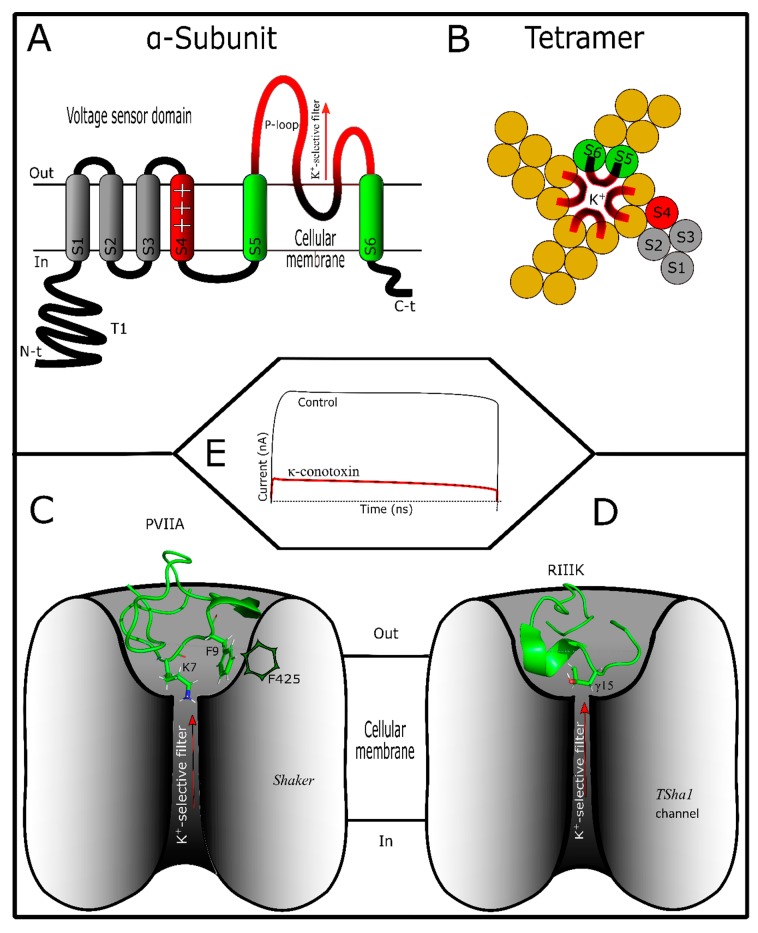
Potassium channels and κ-conotoxin interactions. (**A**) Basic structure of the α-subunit from VGKCs showing segment S4 with positive charges and pore region structured by segments S5 and S6. In this P-loop (highlighted in red) κ-conotoxins interact. Here, accessory subunits are omitted. (**B**) VGKC organization by four α-subunits showing ion pore (P-loops). (**C**) κ-conotoxin PVIIA (PDB: 1AV3) interacting with related Shaker channels. In the dyad model, two amino acids (Lys and Phe) are important for toxin interaction. Lys is inserted in the selective filter of potassium channel. (**D**) RIIIK and related Shaker channel interaction. In this interaction 4-hydroxiproline residue at the 15^th^ position of RIIIK is responsible for the K^+^-flow block. (**E**) Typical electrophysiologic record of K^+^-currents before (black) and after (red) adding the toxin. It can be observed that K^+^-currents decrease after toxin application caused by ion pore obstruction. Here K^+^-current types are not considered. VGKC structures were designed as described by the authors in the text.

Potassium channels are the most abundant and varied ion channels in nature [90]. They are responsible for potassium flow across the membrane, allowing cell excitability to be maintained [91]. Other physiological roles of potassium channels involve cell proliferation, apoptosis and hormone secretion [92]. When potassium channel disturbance occurs, autoimmune, chronic inflammatory and metabolic diseases and cancer can develop [12]. Their fundamental organization is a tetrameric structure of α-subunits, which constitutes the K^+^ selective filter [93]. Sometimes, α-subunits occur together with accessory subunits (i.e., β) depending on the K^+^ channel type [94]. They are grouped in four large families, namely voltage-gated K^+^-channels (K_v_) with 12 subfamilies [95], K^+^-channels activated by calcium (K_ca_) with five subfamilies [96], inwardly rectifying (K_ir_) with seven subfamilies [97], and two-tandem-pore domain K^+^-Channels (K_2P_) with 16 subfamilies [98]. Conotoxins that interact with potassium channels have demonstrated that they are more active in (K_v_) channels [99]. Thus, this section will explain the structure of this channel group (Figure 2A,B). K_v_ channels (VGKCs) are tetramers structured by α-subunits, in which each monomer has six transmembrane segments (S1–S6) [94]. Segments S5 and S6 constitute the ion-selective filter, while segment S4, being positively charged, plays an important role in the channel kinetics [93]. Therefore, voltage sensor S4 segment is responsible for VGKCs’ activity [100,101]. 

Conotoxins that interact with VGKCs are varied in structure, since they are found in various superfamilies (Tables S1–S3). κ-Conotoxins can be disulfide-rich conotoxins shared by A, I, J, M, O superfamilies or conkunitzin-S1, or they can be disulfide-poor conotoxins such as contryphan-Vn [5]. Commonly, the contryphan group possesses a tryptophan or leucine residue in D-configuration and presents variable activity [23]. Among them, contryphan-Vn, a κ-conotoxin with two cysteines, was shown to be active in VGKCs and K_ca_ [102]. Disulfide-rich κ-conotoxins, moreover, show different frameworks (Table S2). This toxin group has some post-translational modifications such as C-terminal amidation, N-terminal pyroglutamylation, γ-carboxylation, hydroxylation, and glycosylation [5]. The last is considered be more frequent in κ-conotoxins than in other conotoxin families [68]. Although its role in κ-conotoxin activity has still not been identified, it is believed that this post-translational modification could increase its half-life in vivo [103]. The role of other post-translational modifications in κ-conotoxins is still unknown.

Disulfide-rich κ-conotoxins have been shown to be preferential blockers [5]. Thus, when interaction occurs, the toxin can decrease K^+^-currents naturally produced by targeted channels without affecting the action of their molecular mechanism (Figure 2E). An exception for this group is BTX [104]. This toxin, from *C. betulinus* venom, showed K^+^-currents increasing in a voltage-dependent manner in K_ca_ channels [104]. κ-Conotoxin blockers, however, interact directly with the pore localized in the extracellular vestibule of VGKCs (Figure 2C,D). Conkunitzin-S1, from *C. striatus* venom, inhibited K^+^-currents from pore-mutated Shaker potassium channels [4]. This toxin was showed to be a specific blocker of mammalian K_v_1.7 [105]. Similarly, other κ-conotoxins such as RIIIJ and RIIIK from *C. radiatus* [106], SIVA from *C. striatus* [107], pl14a from *C. planorbis* [60], ViTx from *C. virgo* [108], sr11a from *C. spurius* [109], and PVIIA from *C. purpurascens* [110] venoms blocked K^+^-currents from K_v_1 and/or related Shaker VGKCs. In this way, it was observed that this toxin group acts in a similar way to other animal toxin blockers [99]. Scorpion K^+^-blockers (KTx), for example, have at least four interaction modes with their targets [111]. Of these, two interaction models have been demonstrated to be similar to the κ-conotoxins’ activity: a dyad and “ring of basic residues” modes. The former was experimentally observed using modeling studies with PVIIA [112,113,114]. A similar interaction between RIIIJ and K_v_1.2 channels was also observed [115]. The second model was observed in the interaction of the RIIIK toxin with *TSha1* channels from rainbow trout (*Onchorhynchus mychiis*) [116,117]. Other interaction modes have not yet been clarified for ViTx and sr11a toxins [108,109] because they do not have dyad or “ring of basic residues” characteristics. In all cases, key amino acids localized in the extracellular pore vestibule of K^+^-channels are necessary for toxin recognition [118]. 

The dyad model is based on two key amino acid residues (basic and aromatic) strategically distributed in the toxin [119]. κ-Conotoxins, such as PVIIA, show this pattern (Figure 2C). Previously, some notable residues in PVIIA were characterized, including Lys7, Phe9, and Phe23, which are important for channel interaction [112]. K7 is inserted in the ion-selective channel pore, physically preventing ion flow, while the aromatic residues make hydrophobic interactions [113]. Mutagenesis in both the toxin and Shaker channel demonstrated that Phe9 from the toxin is more relevant than Phe23 in the interaction with F425 (loop between S5 and S6 segments) from Shaker VGKCs [112,120]. This interaction affinity occurs due to the structural nature adopted by both toxin and K^+^-channels, which is related to the distances between residues, inter- and intramolecularly [118]. As an example of this phenomenon, it has been shown that PVIIA is active in Shaker, but not in K_v_1 channels [120]. This selectivity is based on the structure of each channel subtype. Both VGKC subtypes have one equivalent aromatic residue homologous in interactions with the (Phe9) toxin. Nevertheless, two residues (Thr449 and Asp447) cross-link in *Shaker* channels, favoring Phe23/Phe425 coupling interaction [118]. The natural lack of this cross-linking in K_v_1 and the mutation in Shaker channels (Thr449Tyr) prevent hydrophobic interactions with Phe23 [118,120]. Other interaction points of PVIIA allow toxin stabilization in the *Shaker* channel vestibule [112,118]. Recently, it was shown that the N-terminal and intramolecular hydrogen bond network of PVIIA are important for toxin stabilization in Shaker channel interaction [114]. In conclusion, PVIIA/Shaker channel interaction is due to two strategically localized residues (Lys7 and Phe9) which allow the toxin activity. Lys7 occludes the pore, preventing K^+^-flow, while Phe9 fixes the toxin to the K^+^-channel vestibule (Figure 2C). Other interaction points such as Phe23 or Arg2 are necessary for toxin stabilization [118].

The other model, the “ring of basic residues”, is based on the distribution of basic residues (Arg and/or Lys) along the molecule, which are spatially arranged when a disulfide bond occurs, forming the basic ring [111]. In a similar manner, basic residues of RIIIK electrostatically interact with diverse points of the pore region vestibule from *TSha1* channels, and Glu354 in *TSha1* is the most important [117]. Leu1, Arg10, Lys18, and Arg19 showed their importance for RIIIK activity, and it was demonstrated that there is no aromatic residue typical of the dyad model [116]. A mode of interaction between KTx (scorpion toxins) and VGKCs, for example, could explain this phenomenon. It has been suggested than the electrostatic interaction between the basic ring of the KTx and the potasium-channel disrupts K^+^-flow [111]. However, for this specific case, RIIIK has a post-translational modified residue (4-hydroxiproline at the 15^th^ position, γ15) that interacts with the VGKC, blocking the K^+^-flow [117]. So, it was suggested that the RIIIK basic ring anchors the toxin to the pore vestibule from *TSha1* channels, while its γ15 residue interacts with carbonyl groups localized on the selectivity filter of VGKCs, altering the normal ionic flow (Figure 2D) [117].

## 5. Conotoxins Interacting with Voltage-Gated Sodium Channels

**Figure 3:**
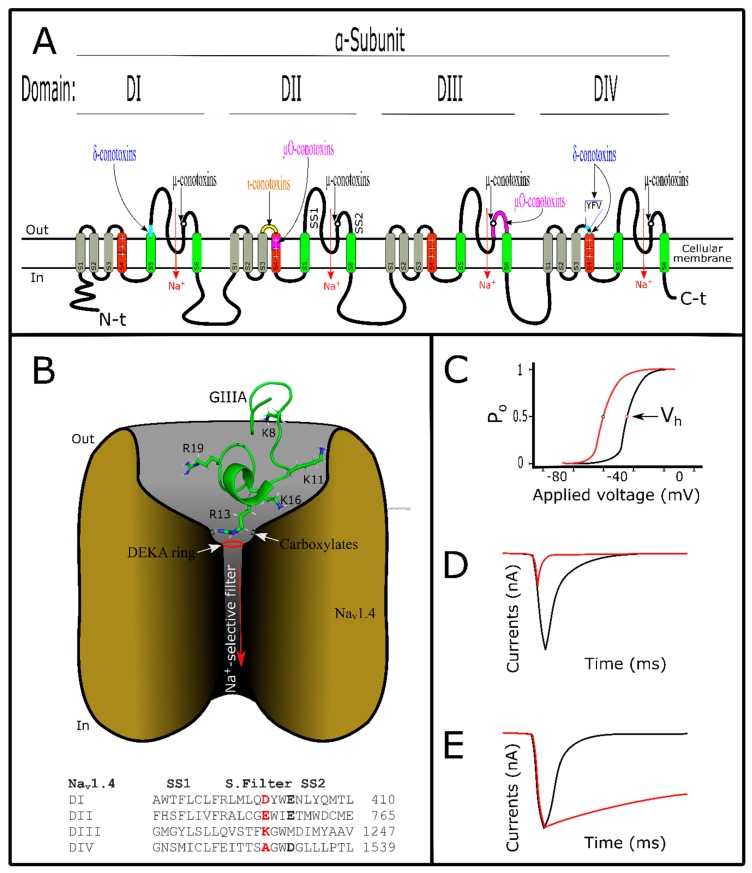
Voltage-gated sodium channels and conotoxins that interact with them. (**A**) Basic structure of the α-subunit of VGSCs. This is constituted by four domains, which compose the Na+ pore. Each domain shows segment S4 with positive charges and pore region structured by segments S5 and S6. Here different interaction localities by conotoxins are showed. Accessory subunits are omitted in this design. (**B**) µ-Conotoxin GIIIA (PDB: 1TCJ) interacting with Na_v_1.4 channel. Arg13 of GIIIA blocks Na^+^-flow by interaction with DEKA ring and outer carboxylates of Na_v_1.4 (showed in the alignment). Other interaction points are described in the text. (**C**) Representative curve of open kinetic states from VGSCs. Maximal Na^+^-current before (black) and after (red) adding the toxin, for each stimulus, with fitted Boltzmann equation. Po is the open probability when a voltage is applied in VGSCs, while V_h_ is the open probability of 50% of these channels. When voltage sensor S4 of domain II is trapped by the ι-conotoxins a voltage shift for hyperpolarized states is observed. (**D**) Typical electrophysiologic record of Na+ currents before (black) and after (red) adding the toxin. It can be observed that Na+ currents decrease after toxin application. (**E**) Typical electrophysiologic record of Na+ currents before (black) and after (red) adding the toxin. Here VGSCs cannot carry out their fast inactivation. Consequently, these keep their open states for more time. VGSC structures were designed as described by the authors in the text.

Voltage-gated sodium channels (VGSCs) are tetrameric structures that allow the sodium ion to pass through the membrane leading to cell depolarization, which is necessary for physiological activity [121]. VGSCs are responsible for starting action potentials in neurons, muscle and immune system cells, among others [91]. These channels could be classified in nine subtypes (Na_v_1.1 – Na_v_1.9) or isoforms, which are distributed among various tissues performing their function [122]. When altered, VGSC subtypes are involved in several diseases such as epilepsy, pain disturbance, autism, and diabetes [14,123]. They are structured by two subunits, the first an α-subunit that composes the ion-selective pore, and the second an accessory β-subunit [121]. The α-subunit is a tetrameric structure made of four domains (DI–DIV), and each domain is structured by six transmembrane segments named (S1–S6) [124,125] (Figure 3A,B). Segments S5 and S6 constitute the Na^+^-selectivity filter of VGSCs, while S4 segments from each domain are voltage sensors responsible for VGSC activity [121,126]. S4 segments from the DII domain are responsible for VGSC activation while S4 segments from the DIV domain are responsible for fast inactivation [121,127]. VGSCs are the target of diverse toxic compounds and each acts on different points of these channels [128]. Depending on the binding site of VGSCs, the toxins exert different effects on them (Figure 3C–E). For example, scorpion toxins are VGSC modulators because they interact with the loops related to the S4 voltage sensors from DII or DIV; thus, they can modulate activation or fast inactivation of VGSCs, respectively [129]. Depending on the superfamily origin, conotoxins act as modulator or blockers of VGSCs (Table 1, Tables S1 and S3).

Conotoxins acting on VGSCs are divided in four families according to their function, and these are µ-, µO-, δ-, and ι-conotoxins [5]. With the exception of µ-conotoxins, the pharmacophore of these toxins is yet to be identified [130], and interaction experiments with their targets are necessary to enhance understanding in this area. These conotoxin groups will therefore be briefly discussed with special emphasis on µ-conotoxins. Conotoxins that target VGSCs show some post-translational modifications. The most common is C-terminal amidation, but there are others that are less frequent, such as pyroglutamate and hydroxyproline [131]. Despite poor knowledge about post-translational modifications, some investigations have been carried out. For example, some amino acids occur with dextrogyre format in natural ι-conotoxins [132,133]. When a laevogyrate format is substituted by natural D-Phe44 in ι-RXIA, its activity is decreased or lost in VGSCs tested [132,133]. Similarly, two toxins close to ι-RXIA (r11a and r11b) were epimerized [132]. However, only one of them decreased its activity in VGSCs [132]. µ-Conotoxins with natural folding show specific activity in their targets. An experiment made with synthetic cysteine isomers of PIIIA, KIIIA, and KIIIB showed activity in VGSCs, but with diverse affinities [134,135], showing that a defined three-dimensional structure is important for toxin/VGSC interaction [25].

ι-Conotoxins are the least studied group. Only two toxins have been identified [33]. ι-LtIIIA, from *C. litteratus*, has six cysteines, exhibiting a type III framework shown to facilitate sodium currents from root ganglion neurons [136]. ι-RXIA and its analogs from *C. radiatus*, with eight cysteines and framework type XI, elicited action potential in amphibian peripheral axons [137]. Curiously, in spite of structural differences, these two toxins showed equivalent activities [132,136,137]. An interaction analysis made with ι-RXIA showed that this toxin can left-shift the voltage-dependent activation from mammal VGSCs [133]. Although not yet full identified for this toxin group, this phenomenon could occur by interaction with S4 of domain DII from VGSCs [138]. Likewise, ι-conotoxins act like β-toxins from scorpions. β-toxins interact with the S3-S4 loop of DII, trapping the S4 movement in pre-open states from VGSCs [124]. Consequently, it will be necessary to apply less energy to activate VGSCs again, and because of this, the voltage is shifted to hyperpolarizing states (Figure 3A,C).

As with ι-conotoxins, it was suggested that µO-conotoxins interacting with the S3-S4 loop of DII trap the S4 movement from VGSCs, but only inhibit Na^+^-conductance [138]. Therefore, members of this conotoxin group are modulators but not blockers (Figure 3A,D). µO-conotoxins are hydrophobic polypeptides stabilized by a six-cysteine ICK-motif [139]. These structural and hydrophobic features are challenging due to their synthesis and later folding [5]. Few µO-conotoxins have been described until now, but MrVIA, MrVIB, and MfVIA have been the best studied [5,33,138]. They have showed blocking, preferentially, Na_v_1.8 subtypes [131]. MrVIA and MrVIB toxins, from *C. marmoreus*, blocked voltage-gated sodium currents from snail neurons [140,141]. MfVIA, from *C. magnificus* and inhibited Na+ currents from human VGSCs [142]. Particularly, it was suggested that loop 2 from the MrVIB structure has some flexibility that allows its interaction with VGSCs [139]. Additionally, by mutagenesis of Na_v_1.2 and Na_v_1.4 channels, MrVIA showed interactions with the SS2 pore loop of DIII in Na_v_1.4 subtypes [143]. Interestingly, MrVIA also interacts with the S4 of DII in VGSCs, therefore also interfering in their activation [144] in a similar way to the activity of β-toxins from scorpion venom. β-toxins could interact with the S3-S4 loop of DII, S2-S3 loop of DII, and SS2 loop of DIII [145,146]. These last interaction points are proposed, anchoring the β-toxin to VGSCs while the interaction with S4 from DII provides its activity [147]. It is possible that, like β-toxins, µO-conotoxins could exert their function by interacting with the S4 loop from DII, while they are anchored to the SS2 pore loop of DIII (Figure 3A). A recent study with MfVIA showed that this conotoxin could interact with voltage sensor points embedded in membrane, generating a voltage shift [148]. On the whole, these findings contribute to the idea that µO-conotoxins could be considered an independent conotoxin family [149]. However, this group is included in the µ-conotoxin group (Table 1), which comprises conotoxin blockers.

Like ι- and µO-conotoxins, δ-conotoxins are VGSC modulators, but they target a different locality from those mentioned regarding other modulators [150,151]. To date, over 22 δ-conotoxins have been described [138]. They have six cysteines with a framework VI/VII pattern stabilized in ICK-motif [5]. Like µO-conotoxins, this toxin group has also been difficult to obtain synthetically and to investigate [5]. This is due to their hydrophobic amino acids distributed along molecules. It was suggested that some of these residues could be important for toxin/VGSCs interaction [138,151]. In fact, three residues (positions 12, 23, and 25) differentiated in δ-CnVIB, δ-CnVIC, and δ-CnVID toxins, respectively, showed selectivity toward mammalian VGSC subtypes [152]. These toxins, from *C. consors*, have residues positioned at (12Ile or 12Phe), (12Phe, 23Phe, and 25Leu), and (25Phe), and they have been seen to be selective to Na_v_1.2, Na_v_1.3 and Na_v_1.4 isoforms, respectively [152]. A previous study made with GmVIA, from *C. gloriamaris*, produced an extended action potential in molluscan neurons [150]. This phenomenon is caused by the modification from fast inactivation in VGSCs, as demonstrated by NgVIA and δ-TxVIA toxins’ activity, from *C. nigropunctatus* and *C. textile*, respectively [153]. Indeed, SVIE, from *C. striatus*, interacted with a hydrophobic triad (Tyr-Phe-Val) present at the S4 of DIV from VGSCs [154]. In this interaction (Figure 3A), δ-conotoxins decreased the fast inactivation process by trapping S4, like the interaction mode of α-toxins from scorpions [154]. Recently, an interaction study made with δ-EVIA and Na_v_1.7 showed that the δ-conotoxin additionally interacted with segment S5 of DI [155]. Identification of one Na^+^-current registered when normal kinetic inactivation of VGSCs is affected is shown in Figure 3E.

Differently to previously mentioned modulators, µ-conotoxins inhibit Na+ currents but block VGSCs [40]. More than 20 µ-conotoxins have been described [138], and this group is the most thoroughly characterized among conotoxins that act on VGSCs [5,130,156]. µ-Conotoxins have different cysteine frameworks depending on their superfamily origin [33]. The most common representatives among them are from the M superfamily with six cysteines and type III or IV frameworks [5]. µT-LtVD, from *C. litteratus*, belongs to the T superfamily and has four cysteines with type V framework [157]. See tables (Table 1 and Tables S1–S3) for structure/function. This toxin group is known for targeting VGSCs sensitive to tetrodotoxin (TTX) or saxitoxin (STX) (Na_v_1.1, Na_v_1.2, Na_v_1.3, Na_v_1.4, Na_v_1.6, and Na_v_1.7), but not for (Na_v_1.5, Na_v_1.8, or Na_v_1.9) mammalian subtypes [158]. They interact with overlap sites for TTX or STX in the filter pore from VGSCs [159,160]. µ-conotoxins’ activity on insensitive TTX- VGSCs has not yet been described [33]. However, some of them were described as promiscuous and also act on VGKCs. μ-SIIIA and μ-PIIIA blocked K_v_1.1 and K_v_1.6 channels [161]. These interactions were identified in in silico studies [162]. Among the VGSCs targeted, µ-conotoxins are more selective toward Na_v_1.4 and Na_v_1.2 subtypes, respectively [5]. µ-Conotoxins have basic amino acid distribution, with one of them in the ~13^th^ position key to blocking VGSCs (Figure 3B) [138,163]. Their net positive charge contributes to electrostatic interaction [164]. This facilitates toxin positioning on the local binding site of VGSCs, independently of the basic amino acid distribution [130]. This basic feature of µ-conotoxins could be attracted by the acidic nature of residues localized on outer pore loops of VGSCs [165]. Carboxylates localized in VGSCs’ outer filter are responsible for Na+ permeation [166] and they are the target of µ-conotoxins [130], thus blocking ion flow (Figure 3A,B,D).

GIIIA, KIIIA, BuIIIB, and PIIIA µ-conotoxins have been studied using computational methods which agreed with experimental data [167]. Various models have been tested in the attempt to research µ-conotoxin/VGSC interactions, using elucidated sodium channel structures from bacteria [168,169]. A successful model, using the Na_v_1.4 subtype created from these basic sodium channel structures, was used [170]. It has led to a better understanding of interactions between µ-conotoxins and VGSCs [167]. To date, GIIIA have been the best characterized (Figure 3B) [130]. Experimentally, this toxin interacted with the four domains of pore vestibule from Na_v_1.4 channels [163]. Specifically, residues localized in the S5–S6 loop of D2 could be important for µ-conotoxin/VGSC stability [171]. Amino acid interactions for GIIIA/Na_v_1.4, such as for Lys8/Asp1248, Lys 11/(Asp 1241 and Asp 1532), Lys16/(Glu758 and Asp 1241), and Arg19/(Asp 762), were found [165,169,170]. Arg13 directly interacted with the selective filter (DEKA ring) and outer carboxylates [130,168], Arg13/(Glu 403, Glu 758, and Asp1532), blocking ion flow [165].

## 6. Conotoxins Interacting with Voltage-Gated Calcium Channels

**Figure 4:**
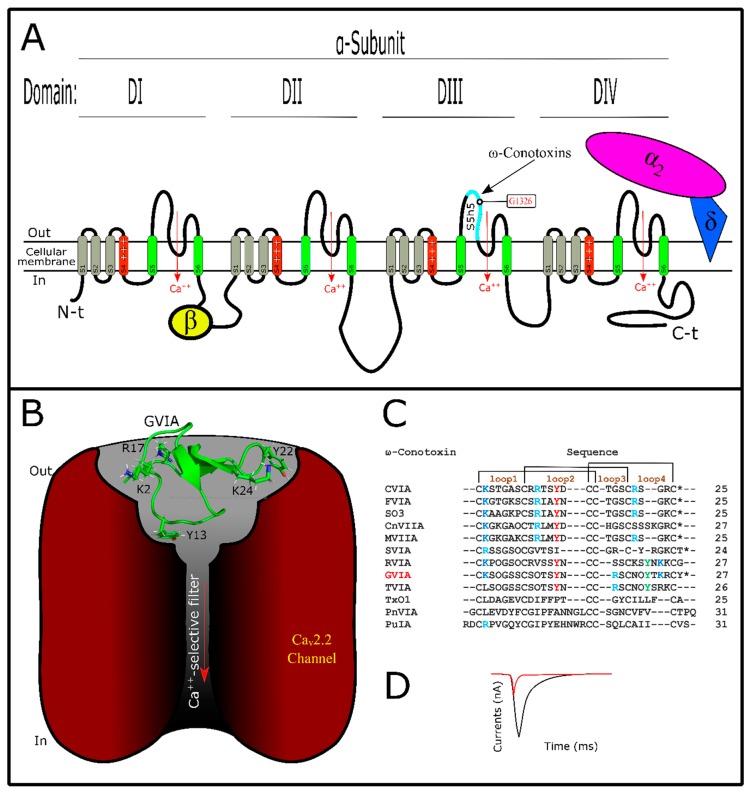
Voltage-gated calcium channels and ω-conotoxin interactions. (**A**) Basic structure of the α-subunit of VGCCs. This is constituted by four domains, which compose the Na+ pore. Each domain shows segment S4 with positive charges and pore region structured by S5 and S6 segments. Here is showed only one known interaction point by ω-conotoxins. Accessory subunits such as β or α_2_δ are showed. (**B**) ω-Conotoxin GVIA (PDB: 2CCO) interacting with Ca_v_2.2 channel. Specific interaction points among them are unknown. (**C**) ω-Conotoxins alignment showing cysteine connectivity and loops. Key amino acids for interaction with their targets are highlighted. * C-terminal amidated, O hydroxyproline. (**D**) Typical electrophysiologic record of Ca++ currents before (black) and after (red) adding the toxin. It can be observed that Ca++ currents decrease after toxin application. Here Ca++ current types are not considered. VGCC structures were designed as described by the authors in the text.

Like sodium channels, voltage-gated calcium channels (VGCC) are tetramers of four domains (DI–DIV) which constitute the α-subunit (Figure 4A,B) [172,173]. Each domain is structured by six transmembrane segments named (S1–S6), and segments S5–S6 are responsible for the ion flow while S4 is positively charged [172,174]. S4 is a voltage sensor and is responsible for opening and closing the channel’s mechanism of action [175]. Furthermore, accessory structures (β, α_2_δ, and γ_1_) can be present, depending on VGCC subtype [174,176]. VGCCs could be involved in multiple physiological functions such as muscle contraction, hormone and neurotransmitter secretion, enzyme activation, etc. [177]. Their specific function can vary with the VGCC subtype. They are classified as (Ca_v_1.1–Ca_v_1.4), (Ca_v_2.1–Ca_v_2.3), and (Ca_v_3.1–Ca_v_3.3) channels [177]. Ca_v_1 and Ca_v_2 groups are sensitive to high voltage, while the Ca_v_3 group is sensitive to low voltage [13]. ω-conotoxins target Ca_v_2.2 channels, and thus this toxin group is responsible for affecting N-type currents [33,178]. Other ω-conotoxins are active in P/Q-type Ca^++^ currents [178,179]. These current types are produced by Ca_v_2.1 channels localized in Purkinge neurons and cerebellar granule cells (P/Q) [176]. Ca_v_2.2 channel subtypes are known as N-type because they are exclusively neuronal and express a Ca^++^ current component that is different from L-type (Ca_v_1.1–Ca_v_1.4) or T-type (Ca_v_3.1−Ca_v_3.3) components [176,179]. Thus, these channel subtypes are involved in nociception more than in any other physiologic process [180]. Interestingly, from of MVIIA, a ω-conotoxin purified from *Conus magus* venom, was developed Prialt^TM^ as drug for the treatment of neuropathic pain [181]. Curiously, this is only conotoxin currently guaranteed by the FDA for use [17].

Like κ- or µ-conotoxins, ω-conotoxins are pore-blockers (Figure 4A,B,D), interacting with the outer vestibule of VGCCs [178]. They belong to the O1 superfamily, sharing a three-dimensional structure with other conotoxins (Table S1) [21]. ω-Conotoxins are structured by six cysteines showing β-sheets in their ICK motif [182,183]. They have a type VI/VII Cys framework (Table S2). This configuration confers on ω-conotoxins four variable inter-cysteine loops that allow their affinity [178]. Other characteristics are their net positive charges [184], which are important in interaction with their targets [178]. Conotoxins, with different features, act on VGCCs and have been described. Contryphan-M, a conotoxin from *C. marmoreus*, has only two cysteines and shows activity in L-type currents [185]. Also, RsXXIVA, from *C. regularis*, has eight cysteines without a defined framework and showed activity in the Ca_v_2.2 channel current [186]. Varied post-translational modifications are not frequent in this conotoxin group (Figure 4C). They show the typical amidated C-terminal found among conotoxins, and some of them have hydroxyproline in their primary sequence [184]. It was detected that substitutions of hydroxyproline by proline did not affect GVIA activity [187]. Studies about the role of post-translational modifications in ω-conotoxins are still needed for more clarification.

Structurally, ω-conotoxins have some key amino acids distributed in their loops [178]. As a principal residue, Tyr13 (loop 2) is determinant in ω-conotoxin/VGCC interactions, while other residues that are not conserved have affected their affinity [188]. Lys2 (loop1), also conserved among ω-conotoxins (Figure 4C), is important for toxin interaction [187,189,190]. Arg17 (or Arg21 depending on the toxin), Tyr22, and Lys24 in loop 4 are related to binding affinity, while Lys or Arg10 (loop 2) could be related to selectivity toward Ca_v_2.1 or Ca_v_2.2, respectively [5]. In contrast, residues localized between the pore region and S5 loop of domain DIII from Ca_v_2.2 have been shown to be important for ω-conotoxins [191]. One residue from this locality, Gly1326, was key for GVIA and MVIIA recognition [192]. GVIA is the most studied ω-conotoxin; however, to date, the interaction point-by-point of ω-conotoxin/VGCC still needs to be elucidated [31]. In comparison with ω-conotoxins, little has been established about VGCC mutations to evaluate toxin/channel interaction. Consequently, molecular dynamics simulations are restricted [5,193], leaving gaps in the knowledge of this approach.

## 7. Conclusions

It was observed that conotoxins and their targets have strategic amino acid residues that determine their interaction. Key residues from conotoxins have been demonstrated to be important because they confer activity and specificity. These findings are of extreme importance. Nevertheless, there are other features involved in the toxin/target interaction. For example, the electrostatic surface could define toxin potency. The electrostatic surface is attributed by characteristics such as cysteine framework and pair-linking, post-translational modifications and amino acid composition. However, as regards conotoxins, studies focusing on these characteristics are still under way. Also, each conotoxin has a special three-dimensional structure (shape) that allows them to fit into their target. There are two possible interaction modes between conotoxins and targets. α-Conotoxins show a full superficial interaction with the target because they are inserted into the binding site. In contrast, conotoxins acting on ion channels have one defined interaction patch. In this case, key amino acids that are important for their interaction are spatially site-directed. 

In spite of conotoxins’ abundance and the structure/function variety, it is curious that only Prialt®, a conotoxin that acts on calcium channels, has been developed as a drug. The promiscuity of conotoxins poses a challenge to their development as future drugs. These findings suggest that the targets’ interaction points are very similar among subtype groups. The conotoxin/specific target interactions are closely related. In general, for any protein/protein interaction process, the three-dimensional electrostatic surface of conotoxins and their specific targets’ contact area must be carefully analyzed because these features are provided by the space distribution of amino acids. This characteristic determines a key–lock effect which leads to harmonized interactions. For some conotoxin families, such as κ-, ι-, δ-, or ω-conotoxins, more investigation into this area is still necessary.

## Figures and Tables

**Table 1:** Conotoxin family classification.

Family	Target and Mode of Action	Reference
α-conotoxins	Inhibitory competitors of nicotinic acetylcholine receptors (nAChR)	[34]
γ-conotoxins	Acting on neuronal pacemaker currents affecting inward cation currents	[35]
δ-conotoxins	Acting on voltage-gated sodium (Na^+^) channel VGSCs, activating and inactivating them	[36]
ε-conotoxins	Acting on G-protein-coupled presynaptic receptors or calcium channels	[37]
ι-conotoxins	Activating VGSCs	[38]
κ-conotoxins	Blocking voltage-gated potassium (K^+^) channel VGKCs	[39]
µ-conotoxins	Blocking VGSCs	[40]
ρ-conotoxins	Inhibitors of alpha1-adrenoreceptors (GPCR)	[41]
σ-conotoxins	Acting on serotonin gated ion channels 5-HT3	[42]
τ-conotoxins	Acting on somatostatin receptors	[43]
χ-conotoxins	Inhibitors of neuronal noradrenaline transporters	[41]
ω-conotoxins	Acting on voltage-gated calcium (Ca^++^) channel VGCCs	[44]

**Table 2:** α-Conotoxin subdivisions with representative conotoxins.

α-CTx	Primary Sequence	Loop Class	Reference
Framework	and Cys pair connectivity 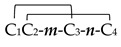	***m/n***	
GI	E**CC**NPA**C**GRHYS**C**GK *	3/5	[55]
ImI	G**CC**SDPR**C**AWR**C** *	4/3	[56]
BuIA	G**CC**STPP**C**AVLYC*	4/4	[57]
AuIB	G**CC**SYPP**C**FATNPD**C** *	4/6	[58]
Vc1.1	G**CC**SDPR**C**NYDHPEI**C** *	4/7	[59]
	Other frameworks		
αJ-pl14a	FPRPRI**C**NLA**C**RAGIGHKYPF**C**H**C**R *	X	[60]
αS-RVIIIA	K**C**NFDK**C**KGTGVYN**C**G(Gla)S**C**S**C**(Gla)GLHS**C**R**C**TYNIGSMKSG**C**A**C**I**C**TYY	X	[61]
αD-VxXXB	DD(Gla)S(Gla)**C**IINTRDSPWGR**CC**RTRM**C**GSM**CC**PRNG**C**T**C**VYHWRRGHG**C**S**C**PG (dimer)	X	[62]

Exclusively, α-conotoxins from the A superfamily have Cys framework I (at the top). *m*/*n* indicates the number of residues among Cys (C_2-3_ and C_3-4_, respectively). Only subclass 3/5 targets muscle nAChRs. * C-terminal amidated, (Gla) γ-carboxyglutamate, (dimer) dimerized molecule, X nonidentified.

**Table 3:** α-Conotoxin activity in diverse nAChR subtypes. Some of the α-conotoxins showed different affinities for homomeric or heteromeric nAChRs or both. nAChRs are arranged from greatest to lowest α-conotoxin activity. nAChR subtypes have the first letter indicating the organism’s origin, such as h for human, m for mouse, and r for rat origins.

α-Conotoxin	nAChR Type Target (IC_50_)	Reference
ArIB	rα7 (1.81 nM) > rα6/α3β2β3 (6.45 nM) > rα3β2 (60.1 nM)	[71]
BuIA	rα6/α3β2 (0.258 nM) > rα6/α3β4 (1.54 nM) > rα3β2 (5.72 nM) > rα3β4 (27.7 nM)	[57]
GIC	hα3β2 (1.1 nM) > hα4β2 (309 nM) > hα3β4 (755 nM)	[72]
GID	rα3β2 (3.1 nM) > rα7 (4.5 nM) > rα4β2 (152 nM)	[73]
ImI	rα7 (220 nM) > rα7 (1.8 μM) > mα1β1γδ (51 μM)hα3β2 (40.8 nM) > hα7 (595 nM)	[74]
Lt1.3	rα3β2 (44.8 nM)	[75]
MII	rα6/α3β2β3 (0.39 nM) > rα3β2 (2.18 nM)	[76]
PeIA	rα9α10 (6.9 nM) > rα6/α3β2β3 (17.2 nM) > rα3β2 (19.2 nM) > rα3β4 (480 nM)	[77]
PnIA	rα3β2 (9.56 nM) > rα7 (252 nM)	[78]
TxIB	rα6/α3β2β3 (28 nM)	[79]
TxID	rα3β4 (12.5 nM) > rα6/α3β4 (94 nM) > rα3β4 (4.5μM) rα3β4 (3.6 nM) > rα6/α3β4 (34 nM)	[80]
Vc1.1	rα3β4 (4.2 μM) > rα3α5β2 (7.2 μM) > rα3β2 (7.3 μM)	[59]

## Supplementary Materials

The following are available online at https://www.mdpi.com/1660-3397/17/6/370/s1, Figure S1: (A) Cysteine connectivity adopts different isomers in α-conotoxins. Structures as described by the authors in the text. (B) Some α-conotoxin alignments showing key amino acid residues highlighted. * C-terminal amidated, O hydroxyproline and γ gamma carboxylic glutamic acid. (C) ACh receptors (-)subunits alignment. Table S1: Conotoxin superfamily classification and families involved. Table S2: Conotoxin Cys framework category and families involved. Table S3: Generic classification and basic structure features from conotoxins.
