# Supplementary material for: Structural and Functional Analyses of Cone Snail Toxins
Source: Mar Drugs. 2019 Jun 21;17(6):370. doi: 10.3390/md17060370 (PMC6628382; doi:10.3390/md17060370)
Supplement: Supplementary file 1 [file marinedrugs-17-00370-s001.pdf]

**Supplementary Materials**

**Table 1.** Conotoxin superfamily classification and families involved.

| Gene Superfamily Name | Families Involved or Target Defined |
|---|---|
| A | α-conotoxins, ι-conotoxins, κ-conotoxins, ϱ-conotoxins |
| B | NMDA receptors |
| B2 | Undefined |
| B3 | α-conotoxins |
| C | α-conotoxins, neurotensin receptors |
| D | α-conotoxins |
| E | Undefined |
| F | Undefined |
| G | Undefined |
| H | Undefined |
| H2 | Undefined |
| I1 | ι-conotoxins |
| I2 | κ-conotoxins |
| I3 | Undefined |
| I4 | Undefined |
| J | α-conotoxins, κ-conotoxins |
| J2 | Undefined |
| K | Undefined |
| L | α-conotoxins |
| M | α-conotoxins, ι-conotoxins, κ-conotoxins, μ-conotoxins |
| M2 | Undefined |

**Table 1.** Conotoxin superfamily classification and families involved, continued.

| Gene Superfamily Name | Families Involved |
|---|---|
| N | Undefined |
| N2 | Undefined |
| O1 | γ-conotoxins, δ-conotoxins, κ-conotoxins, μ-conotoxins, ω-conotoxins |
| O2 | γ-conotoxins |
| O3 | Undefined |
| O4 | Undefined |
| P | Undefined |
| Q | Undefined |
| R | Undefined |
| S | α-conotoxins, σ-conotoxins |
| T | μ-conotoxins, τ-conotoxins, χ-conotoxins |
| U | Undefined |
| V | Undefined |
| W | Undefined |
| Y | Undefined |
| *Ikot-Ikot* | AMPA receptors |
| conoCAP | Undefined |
| Conopressin | Vasopressin/Oxytocin receptors |
| Conkunitzin | κ-conotoxins |
| Conodipine | Like $PLA_2$ |

Taken and modified from Robinson & Norton, 2014 [21]; Himaya & Lewis, 2018 [29]. AMPA: α-amino-3-hydroxy-5-methyl-4-isoxazolepropionic acid receptor, NMDA: N-methyl-D-aspartate receptor, $PLA_2$: phospholipase-$A_2$ activity, Undefined: Putative conotoxins without family or unidentified function.

**Table 2.** Conotoxin Cys framework category and families involved.

| Framework | Cys Pattern | # Cys | Family |
|---|---|---|---|
| I | CC-C-C | 4 | α-, ϱ-, χ-conotoxin |
| II | CCC-C-C-C | 6 | α-conotoxin |
| III | CC-C-C-CC | 6 | α-, κ-, ι-, μ-conotoxin |
| IV | CC-C-C-C-C | 6 | α-, κ-, μ-conotoxin |
| V | CC-CC | 4 | ε-, μ-conotoxin |
| VI/VII | C-C-CC-C-C | 6 | κ-, μ-, γ-, ω-conotoxin |
| VIII | C-C-C-C-C-C-C-C-C-C | 10 | α-, σ-conotoxin |
| IX | C-C-C-C-C-C | 6 | Undefined |
| X | CC-C.[PO]C | 4 | χ-conotoxin |
| XI | C-C-CC-CC-C-C | 8 | κ-, ι-conotoxin |
| XII | C-C-C-C-CCC-C | 8 | κ-, δ-conotoxin |
| XIII | C-C-C-CC-CC-C | 8 | Undefined |
| XIV | C-C-C-C | 4 | α-, κ-conotoxin |
| XV | C-C-CC-C-CC-C | 8 | γ-conotoxin |
| XVI | C-C-CC | 4 | Undefined |
| XVII | C-C-CC-CCC-C | 8 | Undefined |
| XVIII | C-C-CC-CC | 6 | Undefined |
| XIX | C-C-C-CCCC-C-C-C | 10 | Undefined |
| XX | C-CC-C-CC-C-C-C-C | 10 | α-conotoxin |
| XXI | CC-C-C-CCC-C-C-C | 10 | Undefined |
| XXII | C-C-C-C-C-CC-C | 8 | Undefined |
| XXIII | C-C-C-CC-C | 6 | Undefined |
| XXIV | C-CC-C | 4 | Undefined |

| | | | |
|---|---|---|---|
| XXV | C-C-C-C-CC | 6 | Undefined |
| XXVI | C-C-C-C-CCCC | 8 | Undefined |

Taken and modified from Akondi *et al*., 2014[18].

**Table 3.** Generic classification and basic structure features from conotoxins.

| S-family | Family | Structure Involved | | | Target | Mode of Action |
|---|---|---|---|---|---|---|
| | | Cys Framework | Cys Connectivity | Electrostatic Surface | | |
| A | α, | I, II, IV, XIV | ($C_1$-$C_3$, $C_2$-$C_4$ for I and XIV) | TS | nAChRs | Inhibitors |
| | ι, | III | - | UN | VGSCs | Modulators |
| | κ, | IV | - | Patch | VGKCs | Blockers |
| | ϱ | I | ($C_1$-$C_3$, $C_2$-$C_4$) | - | GPCRs | Inhibitors |
| B3 | α | XXIV | - | TS | nAChRs | Inhibitors |
| C | α | - | ($C_1$-$C_2$) | TS | nAChRs | Inhibitors |
| D | α | XX | - | TS | nAChRs | Inhibitors |
| I1 | ι | XI | ($C_1$-$C_4$, $C_2$-$C_6$, $C_3$-$C_7$, $C_5$-$C_8$) | UN | VGSCs | Modulators |
| I2 | κ | XI | - | Patch | VGKCs | Blockers |
| J | α, | XIV | ($C_1$-$C_3$, $C_2$-$C_4$) | TS | nAChRs | Inhibitors |
| | κ | XIV | | Patch | | |
| L | α | XIV | ($C_1$-$C_3$, $C_2$-$C_4$) | TS | nAChRs | Inhibitors |
| M | α, | III | ($C_1$-$C_4$, $C_2$-$C_5$, $C_3$-$C_6$) | TS | nAChRs | Inhibitors |
| | ι, | III | ($C_1$-$C_5$, $C_2$-$C_4$, $C_3$-$C_6$) | UN | VGSCs | Modulators |
| | κ, | III | ($C_1$-$C_4$, $C_2$-$C_5$, $C_3$-$C_6$) | Patch | VGKCs | Blockers |
| | μ | III, IV | ($C_1$-$C_4$, $C_2$-$C_5$, $C_3$-$C_6$) | Patch | VGSCs | Blockers |

| | | | | | | |
|---|---|---|---|---|---|---|
| O1 | γ, | VI/VII | ($C_1$-$C_4$, $C_2$-$C_5$, $C_3$-$C_6$) | - | NPC | (See Table 1) |
| | δ, | VI/VII | ($C_1$-$C_4$, $C_2$-$C_5$, $C_3$-$C_6$) | Patch | VGSCs | Modulators |
| | κ, | VI/VII | ($C_1$-$C_4$, $C_2$-$C_5$, $C_3$-$C_6$) | Patch | VGKCs | Blockers |
| | μ, | VI/VII | ($C_1$-$C_4$, $C_2$-$C_5$, $C_3$-$C_6$) | Patch | VGSCs | Blockers |
| | ω | VI/VII | ($C_1$-$C_4$, $C_2$-$C_5$, $C_3$-$C_6$) | Patch | VGCCs | Blockers |
| O2 | γ | VI/VII, XV | - | - | NPC | (See Table 1) |
| S | α, | VIII | - | TS | nAChRs | Inhibitors |
| | σ | VIII | - | - | SGICh | (See Table 1) |
| T | μ, | V | ($C_1$-$C_3$, $C_2$-$C_4$) | Patch | VGSCs | Blockers |
| | τ, | V | ($C_1$-$C_3$, $C_2$-$C_4$) | - | SR | (See Table 1) |
| | χ | X | ($C_1$-$C_4$, $C_2$-$C_3$) | - | Noradrenaline transporters | Inhibitors |

**TS,** total surface interaction from toxin is important**. Patch,** interaction surface reduced**. UN,** undefined**.** In some cases, such as α- or μ-conotoxins, the contact surface drives the toxin position in the target.

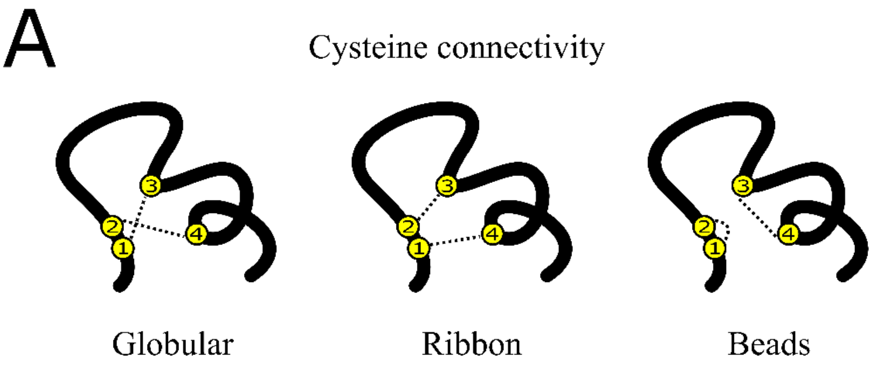


B

| α-Conotoxin | Sequence | |
|---|---|---|
| GIC | ---GCCSHPACAGNNQHIC*--- | 16 |
| PeIA | ---GCCSHPACSVNHPELC*--- | 16 |
| Lt1.3 | ---GCCSHPACSGNNPYFC*--- | 16 |
| TxID | ---GCCSPHVCSAMSPI-C*--- | 15 |
| ArIB | --DECCSNPACRVNNPHVCRRR* | 20 |
| GID | IRDγCCSPNACRVNNOHVC---- | 19 |
| BuIA | ---GCCSTPPCAVLY---C*--- | 13 |
| MII | ---GCCSNPVCHLEHSNLC*--- | 16 |
| ImI | ---GCCSDPRCAWR----C*--- | 12 |
| Vc1.1 | ---GCCSDPRCNYDHPEIC---- | 16 |
| PnIA | ---GCCSLPPCAANNPDYC*--- | 16 |
| TxIB | ---GCCSDPPCRNKHPDLC*--- | 16 |

C

ACh-receptors (-)Subunits alignment

| | 57 | 106 | 108 | 162 | |
|---|---|---|---|---|---|
| Ac-AChBP | RW... | VV | THDGSVMF... | DLSS | 165 |
| (-)hβ2 | EW... | VV | SYDGSIFW... | SLDD | 165 |
| (-)hβ4 | EW... | IV | RSNGSVLW... | SMDD | 165 |

**Figure 1.** Supplementary figure. **A**, cysteine connectivity adopts different isomers in $\alpha$-conotoxins. Structures as described by the authors in the text. **B**, some $\alpha$-conotoxin alignments showing key amino acid residues highlighted. * C-terminal amidated, **O** hydroxyproline and $\gamma$ gamma carboxylic glutamic acid. **C**, ACh receptors (-)subunits alignment.
